# Use of Bayesian networks to dissect the complexity of genetic disease: application to the Genetic Analysis Workshop 17 simulated data

**DOI:** 10.1186/1753-6561-5-S9-S37

**Published:** 2011-11-29

**Authors:** Jia Kang, Wei Zheng, Lun Li, Joon Sang Lee, Xiting Yan, Hongyu Zhao

**Affiliations:** 1 Interdepartmental Program in Computational Biology and Bioinformatics, Yale University, PO Box 208009, New Haven, CT 06520-8114, USA; 2 School of Epidemiology and Public Health, Yale University, New Haven, CT, 06520-8114, USA; 3 Hubei Bioinformatics and Molecular Imaging Key Laboratory, School of Computer Science and Technology, Huazhong University of Science and Technology, Wuhan 430074, China; 4 Keck Biotechnology Resource Laboratory, Yale University, 300 George Street, Room 2119, New Haven, CT 06511, USA

## Abstract

Complex diseases are often the downstream event of a number of risk factors, including both environmental and genetic variables. To better understand the mechanism of disease onset, it is of great interest to systematically investigate the crosstalk among various risk factors. Bayesian networks provide an intuitive graphical interface that captures not only the association but also the conditional independence and dependence structures among the variables, resulting in sparser relationships between risk factors and the disease phenotype than traditional correlation-based methods. In this paper, we apply a Bayesian network to dissect the complex regulatory relationships among disease traits and various risk factors for the Genetic Analysis Workshop 17 simulated data. We use the Bayesian network as a tool for the risk prediction of disease outcome.

## Background

Recent genome-wide association studies have identified many DNA variants (e.g., single-nucleotide polymorphisms [SNPs]) that affect complex human diseases. However, because currently identified genetic variants collectively explain only a small proportion of disease phenotypic variance [1,2], it is important to consider not only genetic factors but also various environmental variables, such as sex, age, and smoking for disease etiology. Therefore it is of great interest to delineate how the complex interactions among the environmental variables, genetic factors, and quantitative traits such as gene expressions lead to disease outcome.

Inferring the dependency structures for multiple interacting quantities is a challenging task, however. Without sophisticated analysis tools, it is difficult to discern conditional independence from dependence of two variables in the data. Bayesian networks are a promising tool for this purpose. First, they provide useful information that describes processes composed of locally interacting components. Second, statistical foundations for learning Bayesian networks from observations and computational algorithms to do so are well developed and have been used successfully in many applications. Finally, although Bayesian networks are mathematically defined strictly in terms of probabilities and conditional independence statements, a connection can be made between this characterization and the notion of direct causal influence [3-6].

By definition, a Bayesian network is a representation of a joint probability distribution, which consists of two components: *E*, which is a directed acyclic graph (DAG) whose vertices correspond to the random variables *X*_1_, …, *X_n_*; and *θ*, which describes a conditional distribution for each variable, given its parents in *E*. Together, these two components specify a unique distribution on *X*_1_, …, *X_n_*. The graph *E* represents conditional independence assumptions that allow the joint distribution to be factorized, economizing the number of parameters. The graph *E* encodes the Markov assumption, which states that each variable *X_i_* is independent of its nondescendants, given its parents in *E*[6].

To fully specify a joint distribution, we also need to specify each of the conditional probabilities in product form. In this paper we treat the variable *X* and its parents *U*_1_, …, *U_K_* as continuous variables, and a natural choice for multivariate continuous distributions is Gaussian distributions. These can be represented in a Bayesian network by using linear Gaussian conditional densities. In this representation the conditional density of *X* given its parents is given by:(1)

When including both quantitative traits and genetic variants in the network analysis, Bayesian networks provide a natural platform for the mining of quantitative trait loci (QTLs). As a result of the small effect size of causal SNPs (mean OR < 1.4 for most common human diseases) and the multiple testing burden, many SNPs identified through genome-wide association studies are false positives if multiple comparisons are not properly taken into account. Because SNPs often exert their effects on quantitative traits, such as gene expressions, which in turn leads to the manifestation of downstream disease phenotypes, the QTL signals are enriched in the true disease causal variants, as suggested by emerging evidence. Therefore QTLs identified for disease-associated quantitative traits are more likely to be true risk factors for the disease and are natural candidates for disease risk prediction.

Functionally, not all SNPs are equally important in causing the disease. Because nonsynonymous SNPs produce a different peptide sequence, they are more likely to be disease causal variants than synonymous SNPs are. Therefore, by incorporating functional annotations of SNPs into the association analysis, we can reduce signal dilution and improve the power of detection of disease variants. In our analysis, we integrate the functional annotation of SNPs by adopting a weighted average approach to generate gene-level scores. We then use data to determine the appropriate weight or contribution of synonymous or nonsynonymous SNPs to the disease phenotype. We present more details in the Methods section.

In this paper, we apply a Bayesian network to dissect the complex regulatory relationships among disease traits and various risk factors for the Genetic Analysis Workshop 17 (GAW17) data and use a Bayesian network as a tool to predict the risk of disease outcome.

## Methods

### Gene-level score derivation

The effective sample size for rare variants is quite small, and association analyses performed at the single-SNP level for these rare SNPs often lack sufficient power. To address this issue, we systematically explored several grouping methods published in the literature for rare variants, including the collapsing method [7], the weighted-sum method [8], the data-adaptive sum method [2], and the kernel method [9]. We found that the well-established weighted-sum method provided solid performance. Therefore we used the weighted-sum method to perform the groupwise analysis for the rare variants.

In the weighted-sum method, the gene-level genetic variable is the sum of minor alleles of all the variants within a particular gene, but each variant is weighted by its minor allele frequency in order to put more emphasis on rare variants.

To incorporate the functional annotation of SNPs into the analysis, for each gene *i*, we obtain two gene level scores *S_i_* and *NS_i_* using only synonymous SNPs and nonsynonymous SNPs, respectively. We then generate a combined gene score:(2)

where *w* is the weight of nonsynonymous SNPs in causing the disease phenotype. Note that *w* is the same for all the genes in the data. Let *P_s_* and *P_ns_* denote the proportions of true positive genes using synonymous and nonsynonymous gene scores, respectively. Then *w* can be estimated by *P_s_*/(*P_s_* + *P_ns_*). For real data, the functional annotation of SNPs can be obtained from public databases such as SIFT. The R package locfdr is used to calculate the proportion of true positive genes.

### Selection of top genes for network construction

To lessen the computational burden, we first perform variable selection to reduce the number of genes to be included in the network analysis. To accomplish this task, we construct simple regression models in which the weighted gene score (described in the previous subsection) and the smoking status are the explanatory variables. For each of the 200 simulated GAW17 replicates, we obtain a list of top genes passing the *p*-value cutoff threshold of 0.1, and those top genes that appear in greater than 100 replicates are retained. Recognizing that replicates may not be available in real data, in a separate analysis we combine 200 replicates into one pooled sample and generate 200 bootstrap samples from the combined sample. The 200 bootstrap samples are then treated as the 200 replicates, and we find that the gene list obtained from the bootstrap approach agrees closely with the genes selected from the replicate-based approach (96% overlap). These steps are repeated for each of the response variables of interest (Q1, Q2, Q4, and disease phenotype), and the union of the marginally associated genes for each response variable is taken, resulting in the selection of 548 genes.

### Network construction

The nodes fed into the Bayesian network contain the following variables: environmental variables (age, sex, smoking status), disease phenotype, quantitative traits (Q1, Q2, Q4), and the gene-level scores for the genes selected in the previously described step.

The conditional likelihood of the variables given their parents is represented in a Bayesian network by using linear Gaussian conditional densities. To avoid biologically uninterpretable directional edges in the network, we ban the following edges from appearing in the network: (1) edges that point from traits (Q1, Q2, Q4, disease outcome) to genes, (2) edges that connect genes to environmental variables, and (3) edges among genes.

### Network optimization

We optimize the Bayesian network using a Monte Carlo Markov chain. The steps are as follows: First, a random network structure using all the variables is initialized. Next, a node from the network is randomly selected. Then, one of the following three operations is performed on the selected node: (1) adding an edge between the selected node and a potential parent node if the selected node has no parents; (2) deleting the edge from an existing parent; or (3) reversing the direction of the edge between the selected node and one of its existing parents. Finally, the post-operational likelihood for the selected node is calculated. To do this, a random number from the uniform distribution (0, 1) is chosen; if the random number is smaller than the Metropolis-Hasting criterion, then the new network configuration is accepted; otherwise, we revert back to the original configuration. After the initialization step, the process is repeated many times until the network likelihood stabilizes.

### Network confidence score derivation

We estimate the confidence of the edges in the constructed Bayesian network by counting the number of times they appear among the 200 replicates. More formally, the confidence score for an edge in the network is calculated as:(3)

where *f*(*G_i_*) = 1 if and only if edge *f* can be extracted from the network constructed from replicate data set *G_i_*. In our analysis, a cutoff of 5 (i.e., edges that appear in at least five replicates) is applied to the confidence score to select the final network. In real data, where replicates may not be available, the confidence score can be obtained from bootstrap samples generated from the original data.

### Disease phenotype prediction

We use half of the data (randomly selected 100 replicates) as the training cohort to obtain a Bayesian network following the steps described earlier. Using the features selected by the Bayesian network, we use a support vector machine to build the risk prediction model in which the response variable is the binary disease outcome and the environmental variables (smoking, sex, age) and the QTLs (which are connected to the quantitative traits in the Bayesian network) are the predictors. The performance of the prediction model is then averaged over the remaining 100 replicates.

## Results

### Network topology

**Figure 1:**
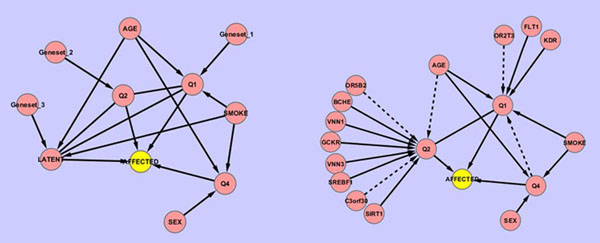
**Bayesian network topologies** (A) The network topology generated from the true simulation model described in the GAW17 answer sheet. (B) The network topology inferred from the data using the Bayesian network approach. Dashed lines indicate false positive edges; solid lines indicate edges that agree with the true simulation model.

In Figure 1 we present the topologies of the Bayesian networks constructed from the true simulation model released in the post-GAW17 solution key and from our approach outlined in the Methods section.

To quantify the advantage of using a joint approach (e.g., Bayesian network) in which multiple traits are considered simultaneously versus a marginal approach (e.g., least absolute shrinkage and selection operator [LASSO]) in which only one trait is considered, we tabulate the area under curve (AUC) value of both methods in Table 1. The calculated AUC values measure how closely the detected genes agree with the true causal genes in the simulation model.

**Table 1:** AUC values of jointly identified QTLs using the Bayesian network and marginally identified QTLs using LASSO

Method	AUC value
Bayesian network	0.61
LASSO	0.57

### Disease phenotype prediction using a Bayesian network

We assess the importance of the functional annotation of SNPs in disease risk prediction by separately building Bayesian networks using only nonsynonymous SNPs and synonymous SNPs. The prediction performance in terms of the AUC value is summarized in Table 2.

**Table 2:** Bayesian-network-based risk prediction performance using SNPs of different functional annotations

Type of SNP used to construct the Bayesian network	Mean AUC value using only genes	Mean AUC value using genes and environmental variables	Mean AUC value using gene and environment variables and quantitative traits
Nonsynonymous only	0.61 ± 0.02	0.83 ± 0.02	0.96 ± 0.01
Synonymous only	0.52 ± 0.02	0.79 ± 0.02	0.95 ± 0.01

## Discussion and conclusions

By examining the regulatory mechanism of genetic factors on various traits, we find that no direct edges in the network connect genes to the disease phenotype. This result suggests that SNPs exert their effects on disease risk indirectly by affecting other quantitative traits that are disease related. This result agrees closely with the true simulation model. In addition, note that in order to most optimally draw inferences about the conditional independence relationships among the nodes in a Bayesian network, we assume that no hidden nodes are missing from the network. However, this assumption is violated in the GAW17 data because there is a latent component of the disease liability that is unobserved. Despite this imperfect setup, the Bayesian network still performs rather well. By comparing the true simulation network to our derived network shown in Figure 1, we observe that most of the relationships among environmental variables and disease or quantitative traits are correctly recovered (denoted by edges with solid lines). Among the eleven gene-trait relationships found in our network, eight of them are true positives and the remaining three genes are moderately but significantly correlated with true causal genes. Furthermore, based on the results summarized in Table 1, it is quite evident that a joint approach in which multiple traits are considered simultaneously (e.g., Bayesian network) has substantial advantages over marginal methods such as the LASSO, in which traits are considered separately.

**Figure 2:**
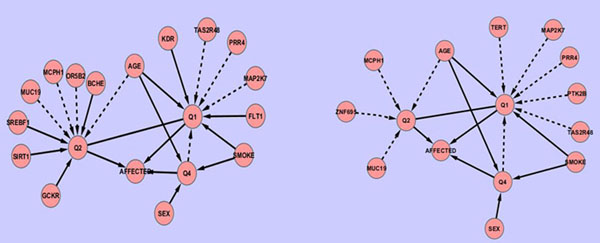
**Bayesian network topologies generated from suboptimal weighting of the functional annotation of SNPs** (A) Network structure when synonymous and nonsynonymous SNPs have the same weight. (B) Network topology inferred using only synonymous SNPs.

Our results suggest that the functional annotation of SNPs should not be overlooked in both association signal detection and disease risk prediction. In our analysis, we estimated the relative contribution of nonsynonymous SNPs versus their synonymous counterparts and found that the disease phenotype is predominantly driven by nonsynonymous SNPs, which closely agrees with the released simulation model. Therefore it is not surprising that the Bayesian network constructed with only synonymous SNPs fails to recover any of the true gene-trait relationships (shown in Figure 2) and yields a much lower AUC value than that from the Bayesian network built with only nonsynonymous SNPs (Table 2).

Finally, we find that genetic variants collectively explain only a small proportion of the disease phenotype. The risk prediction model constructed with only genes as predictors gives an AUC value of only 0.61. However, after the environmental variables are added to the model, the AUC value is dramatically improved to 0.83. This result suggests that although the genetic variants may play an important role in disease etiology, because of their rare nature and because only a small proportion of the population carries these disease variants, their utility as disease risk predictors is limited.

## Competing interests

The authors declare that there are no competing interests.

## Authors’ contributions

JK conceived of Bayesian network study and performed the network based analyses outlined in this paper. WZ, LL, JSL, and XY pre-processed the data and compared different gene score methods. HZ supervised the study.
